# CKAP2L Promotes Esophageal Squamous Cell Carcinoma Progression and Drug-Resistance by Modulating Cell Cycle

**DOI:** 10.1155/2022/2378253

**Published:** 2022-09-02

**Authors:** Wenhu Chen, Yu Wang, Lifang Wang, Hongguang Zhao, Xiaoyan Li

**Affiliations:** ^1^School of Basic Medical Sciences & Forensic Medicine, Hangzhou Medical College, Hangzhou, China; ^2^School of Pharmacy, Hangzhou Medical College, Hangzhou, China; ^3^College of Innovation & Entrepreneurship, Hangzhou Medical College, Hangzhou, China; ^4^Department of Thoracic Surgery, Cancer Hospital of The University of Chinese Academy of Sciences (Zhejiang Cancer Hospital), Hangzhou, China

## Abstract

Esophageal squamous cell carcinoma (ESCC) is one of the most common types of cancer and the leading cause of cancer-related mortality worldwide, especially in Asia. In this study, the gene CKAP2L was selected by GEO, TCGA, and GTEx database analysis. The high expression of CKAP2L is related to the occurrence and development of ESCC. In addition, CKAP2L knockdown can inhibit the growth and migration of ESCC cells, while CKAP2L overexpression has the opposite effect. Furthermore, *in vivo* experiments indicated that down-regulation of CKAP2L can inhibit the tumorigenesis of ESCC cells. KEGG pathway analysis and the STRING database explored the relationship between cell cycle and CKAP2L and verified that depletion of CKAP2L markedly arrested cell cycle in the G2/M phase. Meanwhile, CKAP2L knockdown increased the sensitivity of ESCC cells to flavopiridol, the first CDK inhibitor to be tested in clinical trials, leading to an observable reduction in cell proliferation and an increase in cellular apoptosis. In brief, we identified CKAP2L as a tumor promoter, potential prognostic indicator, and therapeutic target of ESCC, which may play a role in regulating cell cycle progression.

## 1. Introduction

As one of the most common types of cancer, esophageal cancer is one of the leading causes of cancer-associated mortality worldwide [1]. Among them, esophageal squamous cell carcinoma (ESCC) is by far the most common histologic type of esophageal cancer in Asia [2]. In China, there are approximately 470,000 new cases of esophageal cancer each year, which account for more than half of the total worldwide number of cancer diagnoses each year [3]. Overall, its incidence rates are increasing year by year. Despite the development of prophylaxis and early diagnostic techniques, patients were mostly diagnosed with ESCC at an advanced stage and postoperative survival is still poor [4]. Therefore, identifying effective diagnostic and prognostic biomarkers will aid the development of the prevention and cure of ESCC.

CKAP2L (Cytoskeleton-associated protein 2-like) mainly exists in the spindle, spindle pole, and centrosome of the cell, and is an important part of the centrosome of a cell [5–7]. The CKAP2L gene is composed of nine exons and is an indispensable protein for nerve stem or precursor cell division [8]. It plays a crucial role in cell cycle progression and spindle formation during mitosis. Previous studies have found that CKAP2L is highly expressed in multiple malignancies and is always associated with the occurrence, development, and poorer prognosis [9, 10]. However, the role of CKAP2L in ESCC has not been addressed.

In this study, CKAP2L, which may be associated with ESCC, was screened out by the GEO database, and the specificity of CKAP2L in tumors was confirmed by TCGA and GTEX databases. We analyzed the expression of CKAP2L in ESCC tissues and normal tissues, and it was found that CKAP2L was significantly upregulated in ESCC tissues, which was also confirmed in ESCC cells. The function of CKAP2L in ESCC cell proliferation and migration was investigated by constructing KYSE150 and Eca109 cells with silenced and overexpressed CKAP2L, separately. Meanwhile, a synergistic effect was observed in CKAP2L depletion in combination with pan-CDK inhibitor by flavopiridol treatment, which resulted in significant suppression of cell proliferation and the promotion of cellular apoptosis. *In vitro* experiments, mouse xenograft tumors model also confirmed that down-regulation of CKAP2L could inhibit tumor growth, and overexpression of CKAP2L promoted tumor growth. Finally, we aimed to explore the expression, function, and clinical significance of CKAP2L in ESCC, and further reveal the underlying regulatory and drug-resistant mechanism of CKAP2L in promoting ESCC progression.

## 2. Results

### 2.1. Identification of CKAP2L as a Critical Gene That Promotes ESCC Proliferation

**Figure 1:**
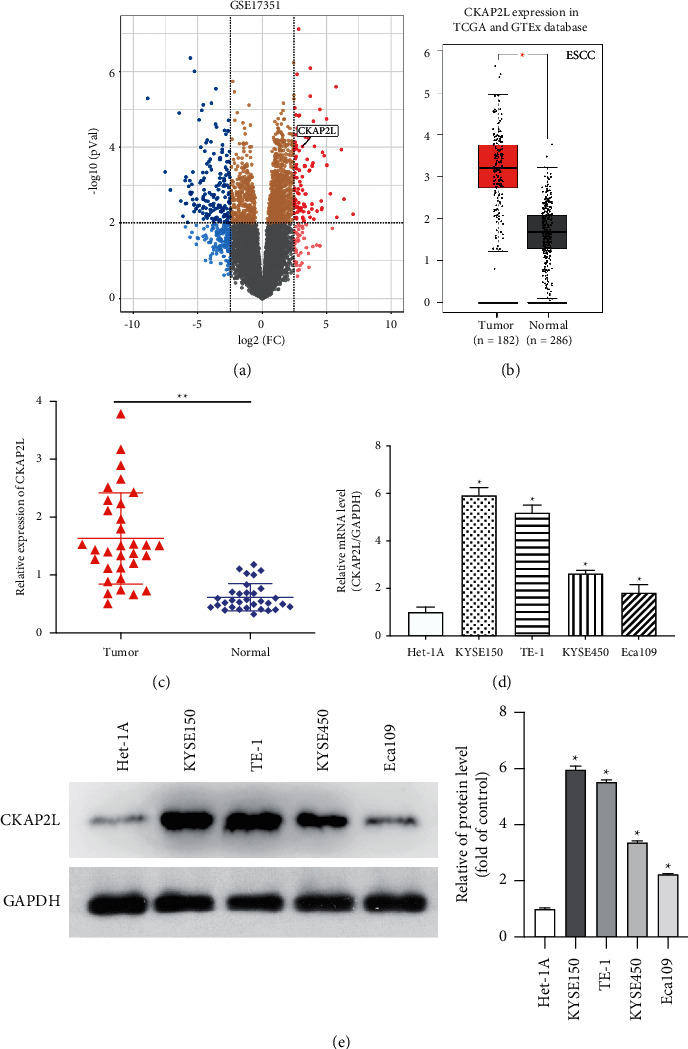
CKAP2L is overexpressed in ESCC tissues and cell lines. (a) Volcano plot for differentially expressed genes in ESCC on the basis of GSE17351. The fold change (FC) of genes was assessed by log transformation. |logFC| >2.5 and adjusted *P* < 0.05 were defined as the screened threshold. (b) The data were from TCGA & GTEx database and analyzed by the GEPIA website, the expression of CKAP2L was upregulated in tumor tissues compared with normal tissues (*P* < 0.05). (c) Expression levels of CKAP2L in clinical samples were detected by RT-qPCR. ^*∗*^*P* < 0.05 by student's *T*-test. (d) Expression levels of CKAP2L in ESCC cell lines were detected by RT-qPCR. ^*∗*^*P* < 0.05 by student's *t*-test versus Het-1A. (e) Western blot analyses the protein expression of CKAP2L in ESCC cells compared with the normal cells Het-1A. GAPDH was used as a loading control in the western blot. The data were expressed as mean ± SD (*n* = 3), ^*∗*^*P* < 0.05.

To investigate the effect of CKAP2L in ESCC, we analyzed the data from the GEO dataset (GSE17351) and discovered CKAP2L expression to be up-regulated in ESCC samples (Figure 1(a)). Coincidentally, we found that CKAP2L was noticeably overexpressed in ESCC tissues compared to normal tissues in TCGA and GTEX databases (Figure 1(b)). Then, ESCC tissues and matched adjacent normal tissues (32 pairs) were collected and detected by RT-qPCR. Consistent with the information in the database, CKAP2L expression was significantly increased in the tumor tissues (*P* < 0.05, Figure 1(c)). It was also shown the expression of CKAP2L at the mRNA level and protein level in four ESCC cell lines (TE-1, Eca109, KYSE150, KYSE450) and an oesophageal squamous epithelial cell line (Het-1A) (Figures 1(d) and 1(e)). Collectively, these data suggest the potential role of CKAP2L as a facilitator during the proceeding of ESCC.

### 2.2. CKAP2L Promotes Cell Proliferation and Migration of ESCC Cells In Vitro

**Figure 2:**
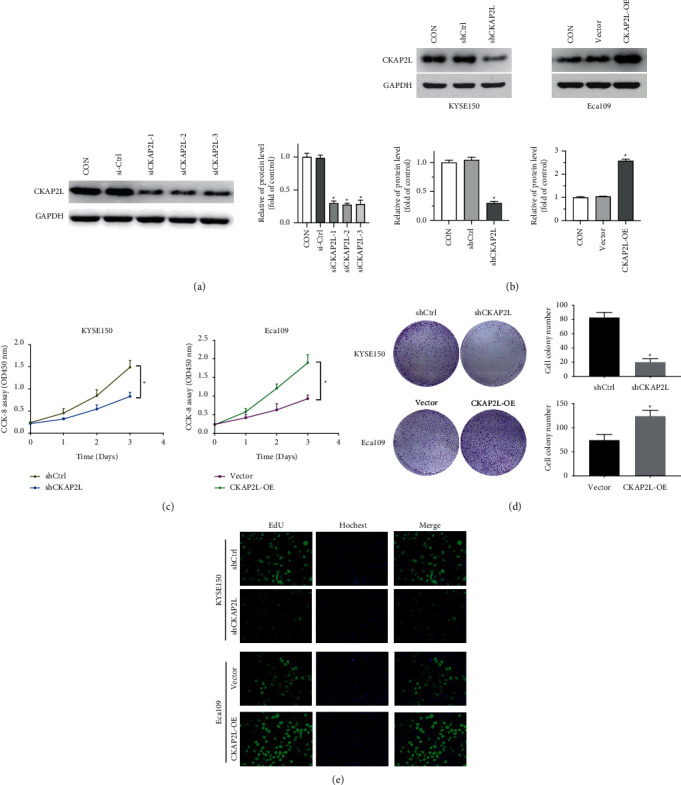
CKAP2L promotes ESCC cell proliferation in vitro. (a) The expression of CKAP2L was knockdown by siRNAs in KYSE150 cells. ^*∗*^*P* < 0.05 by student's *t*-test versus si-Ctrl. (b) Western blotting was used to measure the expression of CKAP2L after knockdown or overexpression by shCKAP2L and CKAP2L-OE. ^*∗*^*P* < 0.05 by student's *t*-test versus shCtrl. (c), (d) CCK-8 and colony formation assays were performed to determine the proliferation ability of ESCC cells transfected with shCKAP2L and CKAP2L-OE. And, the colony number was counted. ^*∗*^*P* < 0.05 by student's *t*-test. (e) EdU analysis displayed that cell proliferation was increase by CKAP2L in ESCC cells. The data were expressed as mean ± SD (*n* = 3), ^*∗*^*P* < 0.05.

To further explore the underlying biological function of CKAP2L in ESCC we transiently depleted CKAP2L expression by siRNAs in KYSE150 cells and the most effective sequence was selected to construct a stable cell line by the lentiviral delivery system (Figure 2(a)). Next, CKAP2L was overexpressed by the transfection of CKAP2L-OE in Eca109 cells. Western blot confirmed that CKAP2L protein level was respectively decreased and increased compared with the control group (Figure 2(b)). CCK-8 assay showed CKAP2L depletion obviously suppressed the growth of KYSE-150 cells. On the other hand, overexpression of CKAP2L promoted cell proliferation in Eca109 (Figure 2(c)). Similar results were obtained by using a colony formation assay (Figure 2(d)). The results of the EdU assay also showed that the cell proliferation rate of KYSE-150 was significantly reduced by shCKAP2L. Meanwhile, up-regulation of CKAP2L could increase the proliferation rate of Eca109 cells.

**Figure 3:**
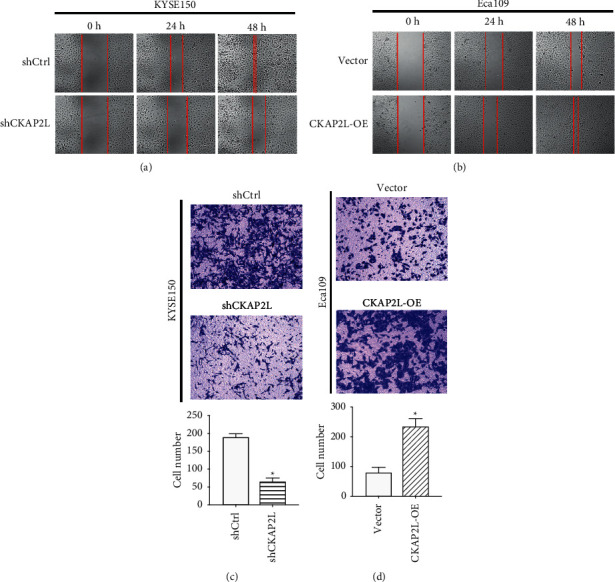
CKAP2L facilitates migration and invasion of ESCC cells in vitro. (a), (b) Wound healing assays were performed to detect the effect of shCKAP2L and CKAP2L-OE on cell-migration ability. (c), (d) The effects of the CKAP2L expression on the invasiveness of KYSE150 and Eca109 cells were investigated in transwell assays. ^*∗*^*P* < 0.05 by student's *t*-test. The data were expressed as mean ± SD (*n* = 3).

Wound healing and transwell assays demonstrated weakened migratory capacity for KYSE150 cells transfected with shCKAP2L. Furthermore, Eca109 cells exhibited a markedly enhanced migratory capacity after overexpression of CKAP2L in both wound healing and transwell assays (Figure 3). Thus, these data showed that CKAP2L significantly promotes the cell proliferation and migration of ESCC cells *in vitro*.

### 2.3. CKAP2L Promotes the Growth of Xenograft Tumors In Vivo

**Figure 4:**
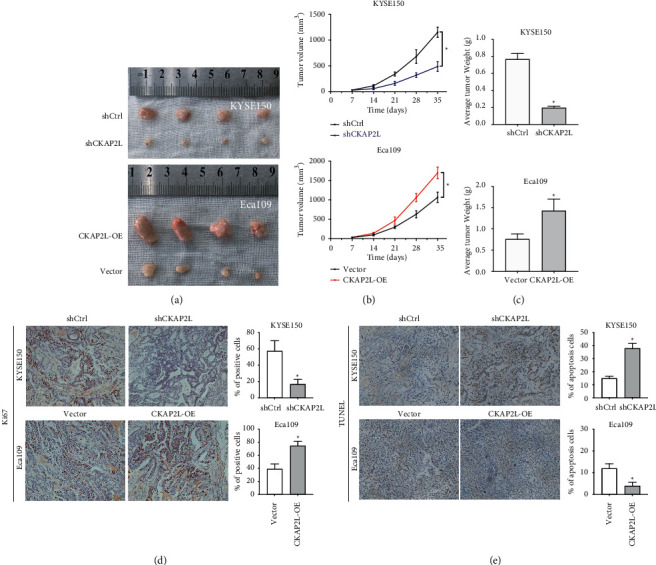
CKAP2L expression positively correlates with the malignant proliferation of ESCC cells in vivo. (a) Effect of subcutaneous injection of KYSE150 and Eca109 cells transfected with shCKAP2L or CKAP2L-OE on the tumor growth (*n* = 4). (b) The mean of tumor diameters for every week is presented ^*∗*^*P* < 0.05 by ANOVA. (c) Quantitative analysis of tumor weight ^*∗*^*P* < 0.05 by student's *t*-test. (d) Immunostaining analysis was performed to measure the positive rate of Ki67 in xenograft tissues. (e) TUNEL assay was performed to determine the rate of cell apoptosis. ^*∗*^*P* < 0.05 by student's *t*-test. The data were expressed as mean ± SD (*n* = 3).

To further confirm the function of CKAP2L on the growth of tumors *in vivo*, KYSE-150 cells with CKAP2L knockdown or Eca109 with CKAP2L overexpression were subcutaneously injected into nude mice to establish a xenograft tumors model. Compared with the shCtrl group, the tumor volume in the shCKAP2L group was obviously reduced, while the tumor volume in the CKAP2L-OE group was larger than in the Vector group (Figures 4(a) and 4(b)). Meanwhile, the average tumor weight of the shCKAP2L group was significantly lower than the shCtrl group, and the CKAP2L-OE group was more than that of the Vector group (Figure 4(c)). In addition, with the technique of histological analysis of tumor sections, we showed that the proliferation activity of tumors by Ki-67 staining was significantly lower in the shCKAP2L group than in the shCtrl group. By contrast, the expressions of Ki-67 in the CKAP2L-OE group were higher than in the Vector group (Figure 4(d)). Meanwhile, the opposite tendency of apoptosis in vivo was also observed with the TUNEL assay (Figure 4(e)). Taken together, these results strongly suggest that CKAP2L plays an important role in the development and progression of ESCC *in vivo*.

### 2.4. CKAP2L Promoted the Overall Progression of ESCC Cell Cycle

**Figure 5:**
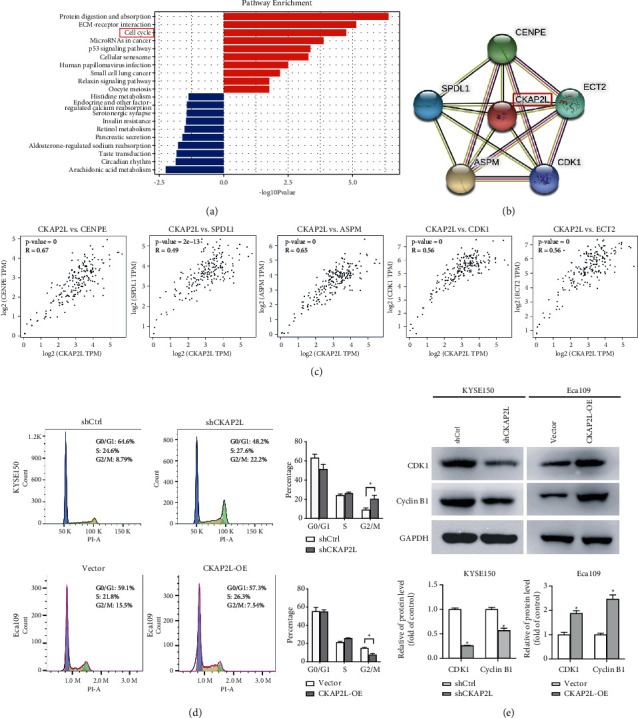
The oncogenic function of CKAP2L in ESCC cells was dependent on cell cycle regulation. (a) KEGG pathway enrichment analysis of differentially expressed genes on GSE17351. (b) Functional protein association network constructed using STRING. (c) The correlation between CKAP2L and cell cycle-related protein expression predicted by the GEPIA database. (d) Effects of shCKAP2L on the cell cycle progression of KYSE150 cells measured by flow cytometric analysis ^*∗*^*P* < 0.05 by student's *t*-test. (e) The expression of Cyclin B1 and CDK1 was analyzed by western blot ^*∗*^*P* < 0.05 by student's *t*-test. The data were expressed as mean ± SD (*n* = 3).

To ascertain the mechanism of oncogenesis of ESCC and to determine the gene expression changes downstream of CKAP2L, KEGG enrichment analysis for differentially expressed genes(DEGs) was performed. The pathways enriched by upregulated DEGs were mainly related to protein digestion and absorption, cell cycle, and p53 signaling pathway (Figure 5(a)). In addition, we also found that CKAP2L proteins interacted with cell cycle-related proteins (CDK1, CENPE, ECT2, SPDL1, ASPM) in the STRING database (Figure 5(b)). Next, the GEPIA database was used to predict the correlation between CKAP2L and above cell cycle-related proteins at the mRNA expression level. The results displayed a dramatic positive correlation between them (Figure 5(c)). To assess the effect of CKAP2L in regulating cell cycle progression, we detected shCKAP2L-induced cell cycling changes in KYSE150 cells using flow cytometry. Down-regulation of CKAP2L induced observable cell cycle arrest at the G2/M phase. Furthermore, overexpression of CKAP2L promoted cell cycle progression at the G2/M phase (Figure 5(d)). Then, we detected the expression of cell cycle-related regulator proteins. After CKAP2L knockdown in ESCC cells, the results of western blot analysis validated that CKAP2L induced the expression of cyclin B1 and CDK1, whereas CKAP2L-OE up-regulated the expression of cyclin B1 and CDK1 in comparison to the Vector group (Figure 5(e)). These results implied that CKAP2L could regulate cell cycle progression, thus contributing to ESCC cell progression.

### 2.5. CKAP2L Depletion Increased Drug Sensitivity of ESCC Cells to Flavopiridol

**Figure 6:**
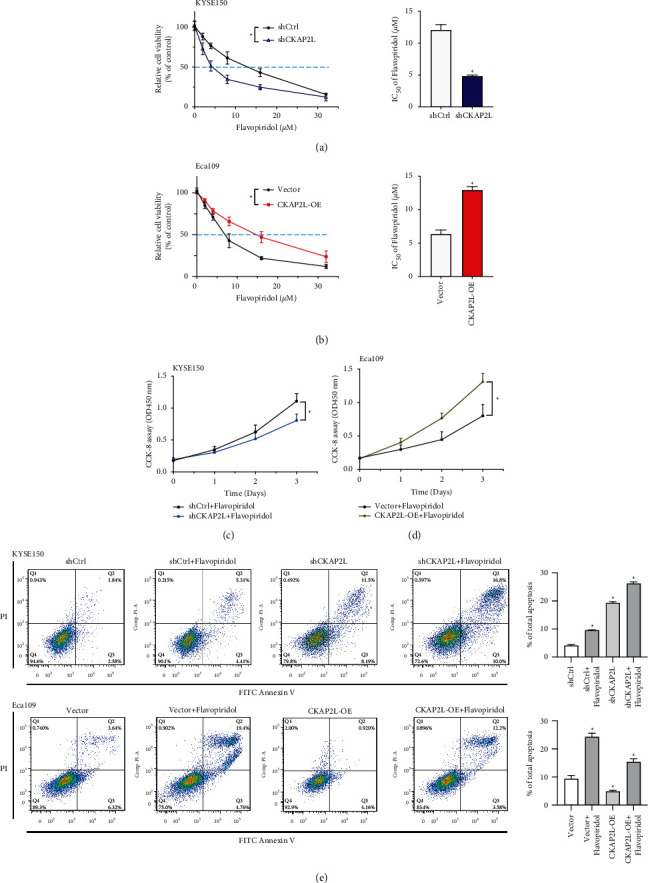
Expression of CKAP2L inhibits the sensitivity of ESCC cells to flavopiridol. (a), (b) IC50 of flavopiridol was determined by CCK-8 assay upon flavopiridol treatment for 24 h in shCKAP2L and CKAP2L-OE transfected KYSE150 and Eca109 cells ^*∗*^*P* < 0.05 by ANOVA and student's *t*-test. (c), (d) CCK-8 assay was used to detect the proliferation activity of shCKAP2L and CKAP2L-OE cells after treatment with flavopiridol. ^*∗*^*P* < 0.05 by ANOVA. (e) Cell apoptosis of shCKAP2L and CKAP2L-OE cells was detected after flavopiridol (5 *μ*M) treatment for 48 h through FITC-Annexin V/PI assays and flow cytometry. The data were expressed as mean ± SD (*n* = 3).

To evaluate the effect of flavopiridol in ESCC cells, a CCK-8 assay was used to assess the sensitivity of KYSE150 and Eca109 to flavopiridol. The intervention of CKAP2L led to a significant decrease in the IC50 of flavopiridol compared with the shCtrl group in KYSE150 cells (shCtrl: 12.07 ± 0.85, shCKAP2L: 4.84 ± 0.16 *μ*M), while the accumulation of CKAP2L elevated the IC_50_ of flavopiridol in Eca109 (Vector: 6.37 ± 0.59, CKAP2L-OE: 12.93 ± 0.53 *μ* M) (Figures 6(a) and 6(b)). Furthermore, CKAP2L depletion in combination with flavopiridol treatment, which resulted in significant suppression of cell proliferation and accelerated cellular apoptosis in KYSE150 cells (Figures 6(c) and 6(d)). The accumulation of CKAP2L promoted cell proliferation and inhibited flavopiridol-mediated apoptosis in Eca109 cells (Figure 6(e)). Collectively, these observations argue that the expression of CKAP2L can alter the drug sensitivity of ESCC cells to flavopiridol.

## 3. Discussion

ESCC is the most frequently occurring subtype of the upper digestive tract esophageal carcinoma. The incidence rate and mortality rate are high [11, 12]. Because of the lack of early clinical symptoms, most of the patients are in the late stage of diagnosis and have a poor prognosis [13–15]. A certain portion of ESCC patients progresses to currently incurable recurrent metastatic cancer [16, 17]. In recent years, with the development of medical technology, although the survival rate of ESCC has improved to a certain extent, it still does not meet the expectations, and it lacks specific biomarkers for early diagnosis [18]. Therefore, it is urgent to explore and find potential targets for the treatment of ESCC.

CKAP2L, which is also called radial fiber and mitotic spindle (Radmis), is located on chromosome 2q14.1 and encodes a 745-amino acid polypeptide [10, 19]. The CKAP2L is validated as a critical constituent of the centrosome, localized to the cytoplasm, mitotic spindle, and spindle pole [20]. Previous studies showed that CKAP2L plays a vital role in regulating cell division in neural progenitor cells, and mutations which are associated with spindle tissue defects, such as mitotic spindle defects and abnormalities in chromosome segregation [21]. Furthermore, loss of function of CKAP2L causes Filippi syndrome and microcephaly [8]. Recent studies have revealed that CKAP2L, an important oncogene, is involved in the biological behavior of many malignant tumors. For example, Wang indicated that abnormal expression of CKAP2L was highly associated with poor prognosis of hepatocellular carcinoma (HCC) and over-expression of CKAP2L promoted migration and invasion in HCC cells [10]. Xiong et al. discovered that up-regulation of CKAP2L in lung adenocarcinoma (LAD) promoted cell proliferation partially by regulating the MAPK signaling pathway, which was also predictive of poor prognosis of LAD patients [9]. Related research reports that CKAP2L could activate the AKT/mTOR signaling pathway in cancer cells and promotes carcinogenesis [22]. Meanwhile, CKAP2L is a vital regulator of miR-4496 activity, and facilitates glioma cell proliferation, migration, invasion, and epithelial-mesenchymal transition [21]. On the other hand, CKAP2L expression increases with the grade of glioma and is associated with a poorer prognosis [23]. However, the role and deep-going regulatory mechanism of CKAP2L in ESCC remains to be further explored.

Initially, we reanalyzed the differential gene expression data from the GEO, TCGA, and GTEx databases and found that CKAP2L is markedly up-regulated in ESCC tissues. Then, it was found that CKAP2L is significantly over-expression in tumor tissues and human cultured cells of ESCC. These results implied that CKAP2L may act as a tumor promoter in ESCC. To reveal the functions of CKAP2L in ESCC, we performed gain- and loss-of-function experiments and observed that treated cells showed an observable accumulation or depletion of CKAP2L protein, consequently resulting in restricted cell proliferation, colony formation, invasion, and migration in both KYSE150 and Eca109 cells. In addition, *in vivo* experiments indicated decreased tumorigenicity after knockdown of CKAP2L, which is consistent with *in vitro* studies. By contrast, up-regulation of CKAP2L increased tumorigenicity. Next, to further elucidate the mechanism of the CKAP2L expression on tumor progression, KEGG enrichment analysis unveiled that the cell cycle pathway plays a vital role in the oncogenesis of ESCC. Meanwhile, PPI network construction was performed and we discovered that CKAP2L proteins interacted with cell cycle-related proteins (CDK1, CENPE, ECT2, SPDL1, ASPM) in the STRING database, and there displayed a significant positive correlation between them in GEPIA database. The abnormality of the cell cycle is the basis of cancer pathogenesis [24, 25]. The cell cycle involves a range of biochemical events, which are mediated by diverse cellular signals [26–28]. Hence, we assessed the effect of CKAP2L knockdown on the cell cycle profile of KYSE150 cells. Flow cytometric analysis showed that the proportion of cells in the G2/M phase was remarkably increased. We also detected cell cycle regulators such as cyclin B1 and CDK1, which were significantly down-regulated at the protein level. Furthermore, CDK inhibitor has become an intense research area for cancer therapy. Flavopiridol is a pan-CDK inhibitor, it has killing effects in a variety of tumors [29, 30]. Our study demonstrated that the expression of CKAP2L can alter the sensitivity to flavopiridol in ESCC cells.

In summary, this study illuminates the function of CKAP2L in promoting the progression of ESCC as well as the potential mechanism. CKAP2L expression was upregulated in ESCC tissues and cell lines, which indicates an oncogenic role of CKAP2L in ESCC. Gain- and loss-of-function experiments displayed that CKAP2L expression level positively regulates the cell proliferation, invasion, and migration of ESCC cells. The arrest of the cell cycle pathway seems to contribute to inhibiting the proliferation and migratory ability of ESCC cells induced by CKAP2L knockdown. CKAP2L knockdown inhibited the progression and drug-resistant of ESCC cells to flavopiridol. Our data suggest that CKAP2L could serve as a potential prognostic and therapeutic target for ESCC.

## 4. Materials and Methods

### 4.1. Analysis of Data from the GEO and TCGA Databases

Microarray datasets (GSE17351) from the GEO database were used to test CKAP2L differential expression for esophageal squamous cell carcinoma [31]. With the goal of improving the reliability of the results, RNA-Seq data from TCGA and GTEx databases were normalized and analyzed from GEPIA (https://gepia.cancer-pku.cn/index.html) to examine the expression level of CKAP2L in ESCC [32].

### 4.2. Tissue Samples and Cell Culture

A total of 32 pairs of ESCC tissue and adjacent normal tissue were acquired from the Thoracic Surgery Department of Zhejiang Cancer Hospital. The tissues were immediately snap frozen in liquid nitrogen until RNA extraction. This study was approved by the Medical Ethics Committee of Hangzhou Medical College. The ESCC cell lines (KYSE150, KYSE450, Eca109, and TE-1) and the human esophageal epithelial cell line (HET-1A) were obtained from the American Type Culture Collection (ATCC). All cells were cultured in RPMI 1640 medium supplemented with 10% fetal bovine serum (Gibco) and 1% penicillin/streptomycin (Invitrogen) in a humidified incubator containing 5% CO_2_ at 37°C. Cells were monitored every 3 days by phase contrast microscopy to ensure that they were in the logarithmic growth phase [33].

### 4.3. Western Blot and RT-qPCR Assays

Total protein was isolated by lysing cells with RIPA buffer (Beyotime, China) and was quantified with a BCA assay kit (Sangon, China). Total protein (50 *μ*g) was separated by electrophoresis (10% SDS-PAGE) and transferred to a PVDF membrane (Millipore, USA). After treatment with a skim milk powder for 2 h at room temperature, the membrane was incubated overnight with a primary antibody reactive with either; CKAP2L (ab221897, Abcam), CDK1 (ab133327, Abcam), Cyclin B1 (4138, CST), or GAPDH (5174, CST) at 4°C. The next day, secondary antibodies conjugated with horseradish peroxidase (HRP) were incubated with the membranes for 2 h. Protein signals were detected with ECL reagents (Invitrogen) [34].

Total RNA was isolated from ESCC tissues and cells using TRIzol reagent (Invitrogen). cDNA synthesis was done with a PrimeScript RT reagent Kit (Takara, Japan). RT-qPCR was performed with SsoFast EvaGreen Supermix (Bio-Rad, USA) and an ABI Prism 7500 Sequence Detection System according to the manufacturer's instructions. The primers used were as follows: CKAP2LF: GGGAAAACTGAAGAGCCAAAACA; CKAP2LR: AGGTTTGACAGGCAAAACAACA;*β*-actin F: TGGCACCCAGCACAATGAA,*β*-actin R: CTAAGTCATAGTCCGCCTAG AAGCA. Relative gene expression was normalized to *β*-actin based on the 2^−ΔΔCt^ method [35].

### 4.4. Transfection of ESCC Cells

Three individual CKAP2L (siCKAP2L-1,-2,-3) and negative control(si-Ctrl) small interfering RNA(siRNA) were designed and purchased from GenePharma Co., Ltd. (Shanghai, China). The most efficient sequence was packaged as lentiviruses (shCKAP2L) by GenePharma. The full sequence of CKAP2L was cloned into the pcDNA 3.1 vector (Invitrogen) to overexpress CKAP2L(CKAP2L-OE). Cell transfection was performed using Lipofectamine 2000 (Invitrogen) as described in the product manual [36].

### 4.5. CCK-8 Assay and EdU Analysis

Cell viability was monitored using the Cell Counting Kit-8 (CCK-8) according to the manufacturer's protocol. ESCC cells were seeded in 96-well plates at a density of 1 × 10^4^/mL. After incubation for 0, 24, 48, and 72 h, 10 *μ*L of CCK-8 solution (Dojindo, Japan) was added to each well and the absorbance at 450 nm was measured using a microplate reader after incubation at 37°C for 2 h. The experiments were repeated in triplicate, independently [37].

An ethynyl deoxyuridine (EdU) was detected using Click-iT Plus EdU Assay Kit (ThermoFisher). Treated cells were observed by fluorescence photo-microscopy with results quantified by counting at least six random fields [38].

### 4.6. Colony Formation Assay

After being treated for 24 h, ESCC cells were placed into six-well plates and cultured with a complete medium for 2 weeks. The medium was replaced every 3 days. Cell colonies were fixed with paraformaldehyde and stained with 0.1% crystal violet for 20 min. Visible colonies were counted for quantification of results [39].

### 4.7. Cell Migration and Invasion Assays

A wound healing assay was performed to assess cell migration. Transfected cells were cultured in 6-well plates to full confluence. Cell monolayers were manually scraped to create wound areas using a pipette tip. Progression of migration was observed and photographed at 0, 24, and 48 h after wounding [39].

Transwell assay was used to evaluate cell invasion. After 24 h of transfection, ESCC cells in an FBS-free medium were placed into the upper chambers that had been pre-coated with Matrigel (Corning, USA), whereas the lower chambers contained a medium with 10% FBS. Following 24 h incubation, the invading cells were fixed with paraformaldehyde and stained with 0.1% crystal violet, and enumerated by light microscopy [40, 41].

### 4.8. Tumor Xenograft Model

Male BALB/c nude mice (4 to 6 weeks old) were purchased from Shanghai SLAC Laboratory Animal Co., Ltd. (Shanghai, China). KYSE150 cells transfected with sh-CKAP2L or CKAP2L-OE were implanted subcutaneously into the flank of mice. Tumor growth was measured every week with a vernier caliper and calculated by using the formula: volume =  (length × width^2^)/2. All the mice were performed euthanasia 5 weeks after inoculation, and their tumor xenografts were removed, weighed, imaged, and subjected to immunohistochemistry for Ki-67 staining [42] and TUNEL assay.

### 4.9. Cell Cycle and Pathway Analysis

In order to reveal the effects of CKAP2L on the cell cycle, we performed flow cytometry. Briefly, transfected KYSE150 cells were cultured in 6-well plates, and then collected and washed with PBS two times and fixed with 70% ethanol at 4°C overnight. Then, cells were treated with RNase A and propidium iodide (PI) according to Cell Cycle Assay Kit (Dojindo, Japan). After incubation, the cells were assayed by flow cytometry (BD, USA). Quantitative cell cycle analysis was conducted using FlowJo software [43].

Pathway annotations of the differential expressed genes were obtained from KEGG and pathway enrichment analysis was performed using DAVID online tools (https://david.ncifcrf.gov/). *P* < 0.01 and FDR <0.01 were selected as the cutoff criteria to identify the enriched pathways. In addition, protein-protein interactions (PPIs) analyses were conducted to construct the functional protein association network, and CKAP2L was used to query the STRING database. Protein interactions with a combined score of >0.4 and a PPI enrichment value <0.01 were considered significant [44].

### 4.10. Cell Apoptosis Analysis

KYSE150 and Eca109 cells were treated with flavopiridol (5 *μ*M) for 48 hours and harvested by trypsinization, the cells were washed twice with PBS and treated with Annexin V-FITC Apoptosis Detection Kit (Dojindo, Japan) according to the manufacturer's instructions. Flow cytometry was used to detect stained cells and analyze the level of apoptosis.

### 4.11. Statistical Analysis

All data were analyzed with GraphPad Prism 7.0 software and presented as mean ± SD. Statistical significance was determined with a *t*‐test or a one‐way analysis of variance (ANOVA) test. Differences were considered statistically significant at *P* < 0.05.

## Data Availability

The authors declare that all data supporting the findings of this study are available within the article.
